# Identification and validation of a pyroptosis-related gene signature in intracranial aneurysm

**DOI:** 10.1515/biol-2025-1243

**Published:** 2026-05-07

**Authors:** Yang Liu, Yuanchi Cheng, Qian Li, Delong Wang

**Affiliations:** Department of Neurology, Minhang Hospital, Fudan University, Shanghai, China; Institute of Science and Technology for Brain-Inspired Intelligence (ISTBI), Fudan University, Shanghai, China; Department of Neurosurgery, Shanghai Jiao Tong University Affiliated Sixth People’s Hospital South Campus

**Keywords:** intracranial aneurysm (IA), pyroptosis, immune infiltration, caspase 8, inflammation

## Abstract

Intracranial aneurysm (IA) is a life-threatening cerebrovascular disorder, the underlying molecular mechanisms of which remain incompletely elucidated. Pyroptosis, a pro-inflammatory form of programmed cell death, has been implicated in various vascular pathologies. However, its role in the pathogenesis of IA remains largely unclear. In this study, we identified and validated a pyroptosis-related gene signature linked to IA using GEO dataset. Differential expression analysis and pathway enrichment methods revealed key pyroptosis-related markers, including CASP8, NOD2, PYCARD, which were strongly associated with IA progression. Functional analysis highlighted their involvement in inflammatory and immune pathways, particularly in promoting vascular remodeling through pyroptosis-driven mechanisms. Machine learning approaches refined these markers into a robust predictive signature. Validation in independent cohorts confirmed their diagnostic and prognostic potential for IA. Moreover, we validated the upregulation of these genes and found the activation of CASP8 in clinical IA samples. Our findings provide novel insights into the molecular underpinnings of IA, offering a framework for the development of pyroptosis-based biomarkers and therapeutic strategies aimed at early detection and targeted intervention in IA management.

## 1 Introduction

Intracranial aneurysm (IA) is a cerebrovascular disorder characterized by localized dilation of the arterial wall due to structural weakness. It is a major cause of subarachnoid hemorrhage (SAH), which carries high morbidity and mortality rates [1], 2]. The pathogenesis of IA is complex and involves multiple factors, including genetic predisposition, hemodynamic stress, inflammatory responses, and vascular remodeling [3], [4], [5]. Despite advancements in neuroimaging and surgical interventions, the early detection and prevention of IA rupture remain significant challenges [6], 7].

Inflammation plays a crucial role in IA formation and progression. Studies have shown that inflammatory cell infiltration, particularly macrophages and neutrophils, contributes to endothelial dysfunction and extracellular matrix degradation, ultimately leading to aneurysmal growth and rupture [8], [9], [10]. Various inflammatory pathways, such as the NF-κB and NLRP3 inflammasome pathways, have been implicated in IA pathophysiology [11], [12], [13]. However, the specific mechanisms linking inflammation to IA progression remain incompletely understood.

Pyroptosis, a form of programmed inflammatory cell death mediated by inflammasomes and pyroptosis-related genes (PRGs), has recently emerged as a key regulator of vascular inflammation [14], [15], [16]. Unlike apoptosis, pyroptosis is characterized by cell membrane rupture and the release of pro-inflammatory cytokines and alarmins, which exacerbate tissue damage and promote a pro-inflammatory microenvironment [17]. Pyroptosis has been implicated in various cardiovascular and cerebrovascular diseases, including atherosclerosis and ischemic stroke, but its precise role in IA remains largely unexplored [18]. Given the inflammatory nature of IA, understanding the involvement of pyroptosis in aneurysm pathogenesis may provide novel insights into disease progression and potential therapeutic targets.

Current studies on IA primarily focus on genetic risk factors, hemodynamic forces, and general inflammatory responses, but few have systematically investigated the contribution of pyroptosis to IA development. Moreover, while some differentially expressed genes (DEGs) have been identified in IA through transcriptomic studies, a comprehensive pyroptosis-related gene signature and its diagnostic or prognostic relevance remain undetermined [19], 20].

To address this gap, we aimed to systematically investigate the involvement of pyroptosis in IA using bioinformatics and machine learning approaches. We identified differentially expressed PRGs and performed pathway enrichment analyses to elucidate their roles in IA progression. Key PRGs, including CASP8, NOD2, and PYCARD, were further refined into a predictive signature, which was validated in independent cohorts. Additionally, we explored the immune microenvironment of IA and assessed the correlation between PRGs and immune cell infiltration, providing novel insights into the interplay between pyroptosis and IA-associated inflammation. The upregulation of key PRGs were verified by qPCR. We also found strong caspase 8 activation in IA samples.

By elucidating the molecular basis of IA through a pyroptosis-focused approach, our study provides a framework for developing PRG-based biomarkers and targeted therapeutic strategies. Understanding the role of pyroptosis in IA pathogenesis may offer new opportunities for early detection and intervention, ultimately improving patient outcomes.

## 2 Materials and methods

### 2.1 Data acquisition

We retrieved gene expression data for intracranial aneurysm (IA) from the Gene Expression Omnibus (GEO) database (GSE54083) [21]. This dataset includes both IA and control samples. The data underwent preprocessing steps to remove low-quality or outlier samples. Data normalization was performed using the limma package in R (version 4.0.3) [22], specifically utilizing the voom method to account for heteroscedasticity in gene expression data. Log2 transformations were applied to the expression values to stabilize variance and improve interpretability. The quality of the data was assessed by visualizing sample distribution with principal component analysis (PCA) and checking for batch effects using the sva package in R.

### 2.2 Identification of PRGs

A list of pyroptosis-related genes (PRGs) was compiled from public databases, including GeneCards (https://www.genecards.org/) and published literature [23]. The genes included in this list are implicated in pyroptosis through inflammasome activation. Differential expression analysis between IA and control samples was performed using the limma package [22] in R. Genes were considered differentially expressed if they met the following criteria: false discovery rate (FDR) < 0.05 and |log2 fold change (log2FC)| > 1.

### 2.3 Differentially expressed genes and volcano plot

Differentially expressed genes (DEGs) were analyzed using the limma package to compare IA samples and control samples. A volcano plot was created to visualize significant DEGs, applying a p-value threshold of <0.05 and requiring a |log2 fold change| > 1 for gene selection. The plot was generated using the ggplot2 [24] R package to illustrate the differential expression of key genes.

### 2.4 Pathway enrichment and functional analysis

Functional enrichment analysis was performed using the clusterProfiler [25] package in R. Gene Ontology (GO) and Kyoto Encyclopedia of Genes and Genomes (KEGG) pathway enrichment analyses were performed to investigate the biological functions associated with the identified PRGs. The top pathways with an adjusted p-value <0.05 were considered significantly enriched. Gene set enrichment analysis [26] (GSEA) was also performed to explore differences between IA and control groups, with a focus on immune-related pathways.

### 2.5 Machine learning model development

Machine learning techniques were employed to refine the PRG signature into a robust predictive model. The caret package in R was used to train a random forest model with the top 10 differentially expressed PRGs as input features. Cross-validation using 10-folds was applied to evaluate model performance. The model’s diagnostic ability was assessed using receiver operating characteristic (ROC) curve analysis, and the area under the curve (AUC) was calculated to evaluate its predictive accuracy.

### 2.6 Immune cell infiltration analysis

Immune cell infiltration levels were estimated using the CIBERSORT [27] algorithm, which utilizes gene expression data to estimate the proportions of 22 immune cell types in the samples. Immune cell proportions were compared between high-risk and low-risk IA groups. Correlation analysis between the expression of PRGs and immune cell infiltration was performed to explore the relationship between pyroptosis and the immune microenvironment in IA.

### 2.7 Correlation analysis

Pearson’s correlation coefficient was calculated to assess the relationship between the expression of key PRGs (CASP8, NOD2, PYCARD) and the proportions of immune cell types. The correlation results were visualized with heatmaps, generated using the ggplot2 package in R, to show the strength and direction of associations between gene expression and immune cell types.

### 2.8 Validation and independent cohort analysis

The predictive PRG signature was validated using independent IA cohorts from GSE75436 and GSE122897 [28]. Diagnostic and prognostic validation was carried out by comparing the PRG signature’s performance in distinguishing high-risk IA patients from low-risk controls. The model’s performance was assessed with ROC analysis to verify its reliability as a diagnostic and prognostic tool.

### 2.9 Statistical analysis

All statistical analyses were performed using R software (version 4.0.3). Differential expression analysis was conducted using the limma package. Differential expression analysis was carried out using the limma package. Pathway enrichment analysis was conducted using clusterProfiler, and immune cell infiltration analysis was performed using the CIBERSORT package. The machine learning model was evaluated using 10-fold cross-validation and ROC curve analysis. Statistical significance was defined as a p-value <0.05, and all tests were two-tailed. Pearson’s correlation coefficient was used to analyze the relationships between gene expression and immune cell types. All statistical methods were carefully chosen to ensure reproducibility and reliability of the results.

### 2.10 Preparation of samples with IAs and STAs

Clinical samples were obtained from eight patients at the Department of Neurology, Minhang Hospital, Fudan University (Shanghai, China). Among them, four samples were collected from patients diagnosed with intracranial aneurysms (IAs) based on digital subtraction angiography or computed tomography angiography (CTA), and who subsequently underwent craniotomy and aneurysm clipping surgery. In addition, four samples of superficial temporal arteries (STAs) were collected as control tissues. These STA samples were obtained from patients undergoing neurosurgical procedures for non-aneurysmal conditions such as craniocerebral trauma or intracranial tumors, and the absence of IAs was confirmed by CTA. Written informed consent was obtained from all participants prior to sample collection.

Total RNA and protein were extracted from all tissue samples using standard protocols. For RNA extraction, samples were processed using TRIzol reagent (Invitrogen, USA), followed by RNA quality and concentration assessment with a Nanodrop spectrophotometer. For protein extraction, tissues were lysed using RIPA buffer supplemented with protease inhibitors (Beyotime, China). The extracted RNA and proteins were subsequently used for gene expression analysis and Western blotting, respectively.

**Informed consent:** Informed consent has been obtained from all individuals included in this study.

**Ethical approval:** The research related to human use has been complied with all the relevant national regulations, institutional policies and in accordance with the tenets of the Helsinki Declaration, and has been approved by the Ethics Committee of Minhang Hospital.

### 2.11 Western blot and quantitative real-time PCR of samples

Western blot analysis was performed to detect CASP8 protein expression in IA and STA tissue samples. Protein were separated by SDS-PAGE and transferred onto PVDF membranes (Millipore, USA). Recombinant human caspase 8 (sigma, # C1099) was also loaded as positive control. The membranes were blocked with 5 % non-fat milk in TBST for 1 h at room temperature, followed by incubation overnight at 4 °C with a primary antibody against CASP8 (R&D Systems, # MAB704, 1:1,000). After washing, membranes were incubated with HRP-conjugated secondary antibodies (Cell Signaling Technology, USA) for 1 h at room temperature. Protein bands were visualized using enhanced chemiluminescence (ECL) reagents (Thermo Fisher Scientific, USA).

For quantitative real-time PCR (qPCR), total RNA was extracted from tissue samples using TRIzol reagent (Invitrogen, USA), and cDNA was synthesized using the PrimeScript RT reagent kit (Takara, Japan) according to the manufacturer’s instructions. qPCR was performed using TB Green Premix Ex Taq II (Takara, Japan) on a QuantStudio 5 Real-Time PCR System (Applied Biosystems, USA). The relative mRNA expression levels of CASP8, PYCARD, and NOD2 were analyzed using the 2^−ΔΔCt^ method and normalized to GAPDH as an internal control.

## 3 Results

### 3.1 Identification of PRGs in IA

**Figure 1:**
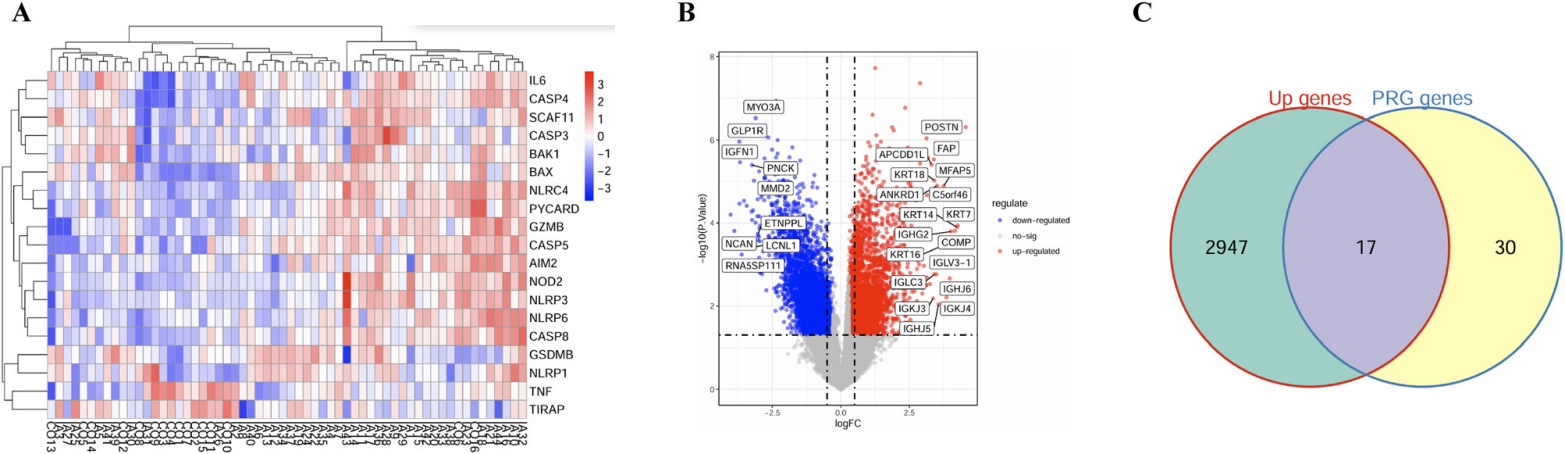
Identification of pyroptosis-related genes (PRGs) in IA. (A) Heatmap displaying the expression of key PRGs (CASP8, PYCARD, NOD2) in IA versus control samples, showing significant differences. (B) Volcano plot highlighting differentially expressed genes (DEGs) with red and blue dots for upregulated and downregulated genes, respectively. (C) Venn diagram showing overlap between upregulated genes and PRGs, identifying 17 common genes for further study.

To explore the role of PRGs in IA, we conducted a differential expression analysis between aneurysm tissues and normal tissues. From the analysis, we identified a set of upregulated and downregulated genes, represented through heatmap clustering (Figure 1A). Notably, the upregulated genes included inflammation-related markers such as IL6, GZMB, CASP5, and NLRC4, which suggest their potential involvement in IA pathogenesis. To further characterize these genes, we performed a volcano plot analysis (Figure 1B) highlighting the most significant upregulated (red) and downregulated (blue) genes. Among these, genes like POSTN and FAP were significantly upregulated, while genes like MYO3A and GLP1R were notably downregulated. This suggests a complex regulatory landscape of gene expression in IA tissues, with inflammatory and fibrosis-related genes playing central roles. Additionally, a Venn diagram (Figure 1C) revealed the overlap between the upregulated genes and PRGs, identifying 17 common genes (IL6 GZMB CASP5 NOD2 NLRC4 NLRP6 AIM2 BAX, etc.). These intersecting genes represent key candidates for further investigation to understand their contributions to IA via pyroptosis mechanisms. Specifically, we aim to focus on these shared genes to unravel their functional roles in IA progression and their potential as therapeutic targets.

### 3.2 Functional enrichment and pathway analysis of PRGs in IA

**Figure 2:**
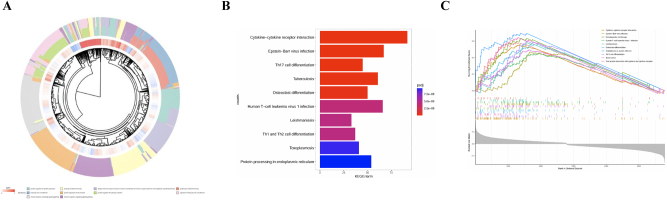
Pathway enrichment of differentially expressed PRGs. (A) Circular plot illustrating enrichment of immune-related pathways like cytokine-cytokine receptor interactions and Epstein-Barr virus infection. (B) Bar plot showing top KEGG pathways enriched in IA, including T-helper cell differentiation and tuberculosis. (C) GSEA plot highlighting immune-related pathway enrichment in IA samples, emphasizing immune dysregulation.

To explore the biological significance of PRGs in IA, we conducted GO and KEGG enrichment analyses. The radial plot (Figure 2A) highlights the enrichment of immune-related terms such as “positive regulation of cytokine production” and “leukocyte-cell adhesion,” underscoring the role of immune response in IA. KEGG analysis (Figure 2B) revealed significant pathways including “cytokine-cytokine receptor interaction” and “Th17 cell differentiation,” emphasizing immune system dysregulation. GSEA (Figure 2C) further supported these findings, showing consistent enrichment in immune pathways. These results suggest that targeting immune responses and pyroptosis may offer therapeutic potential in IA.

### 3.3 Network analysis of PRGs in IA

**Figure 3:**
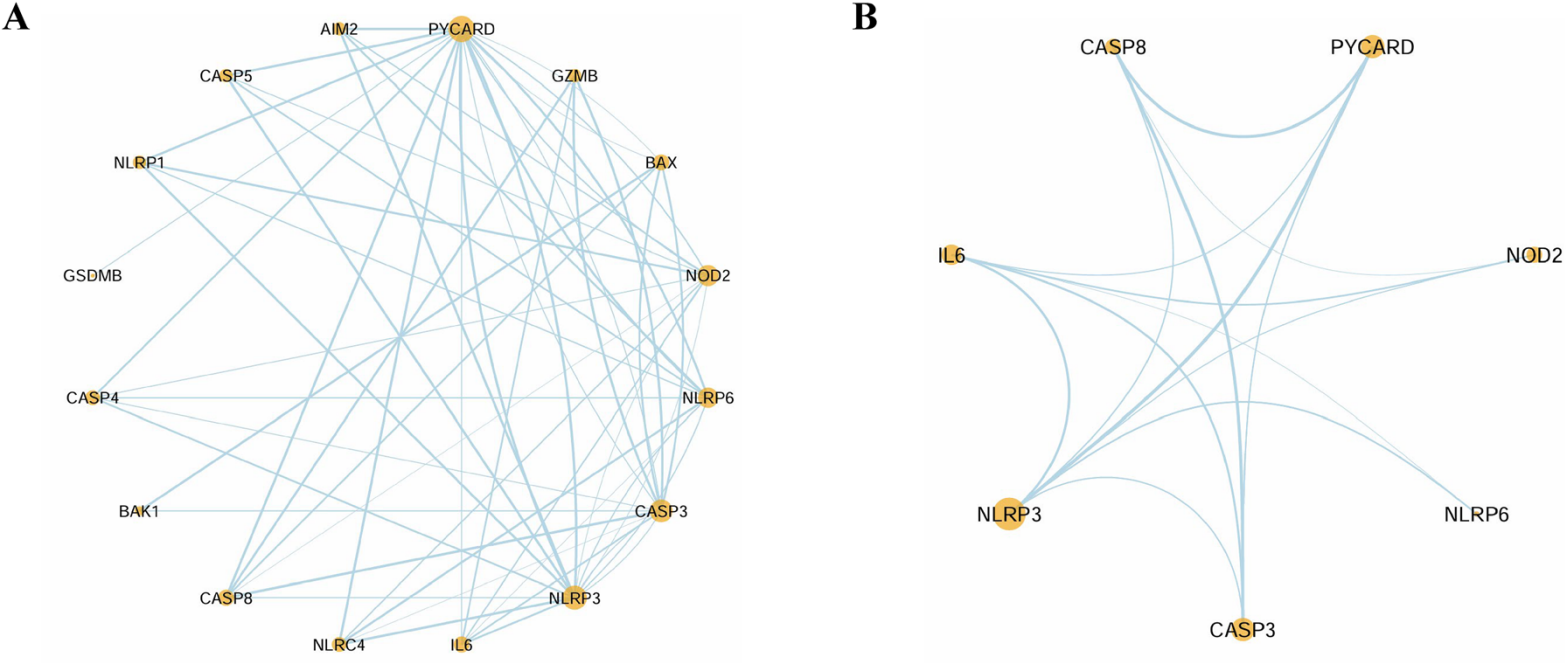
Protein-protein interaction (PPI) network of PRGs in IA. (A) Full PPI network showing interactions among key PRGs (NLRP3, CASP3, IL6), with central nodes linking to other PRGs. (B) Core subnetwork focusing on the critical genes (NLRP3, CASP3, IL6, PYCARD), underscoring their role in IA progression.

A protein-protein interaction (PPI) network was constructed to analyze the regulatory roles of PRGs in IA. The full network (Figure 3A) identified NLRP3, CASP3, and IL6 as key hubs, with dense interconnectivity indicating their roles in inflammation and cell death pathways central to IA pathogenesis. A core subnetwork (Figure 3B) was extracted based on connectivity and functional relevance, highlighting NLRP3 as the central regulator interacting with CASP3, IL6, CASP8, NOD2, and PYCARD. These genes, crucial for pyroptosis and inflammatory processes, underscore NLRP3’s role in amplifying inflammatory responses in IA. This analysis simplifies the complexity of the full network and identifies potential therapeutic targets, emphasizing the central role of NLRP3 and its interactors in IA progression.

### 3.4 Construction of a pyroptosis-related gene signature in IA

**Figure 4:**
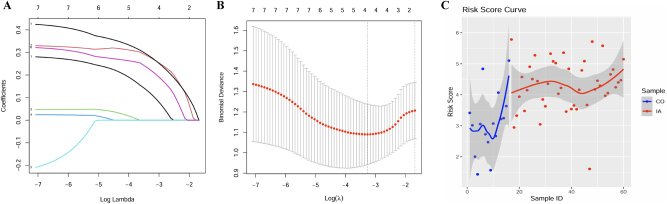
Development and validation of IA risk stratification model. (A) Lasso regression plot showing PRG coefficients for the IA risk prediction model. (B) Cross-validation plot to determine the optimal log(*λ*) value for the gene signature. (C) Risk score curve showing higher risk scores in high-risk IA samples compared to low-risk controls.

To construct a predictive pyroptosis-related gene signature for IA, we performed LASSO regression analysis. The coefficient trajectory plot (Figure 4A) illustrates the selection of essential genes as the penalty parameter (log Lambda) increases, retaining only the most predictive genes for model development.

Using 10-fold cross-validation, the binomial deviance curve (Figure 4B) identified the optimal Lambda value, ensuring a balance between model complexity and accuracy. This approach maximized the predictive performance while minimizing overfitting.

The risk score model, based on the selected gene signature, was applied to IA and control samples. The risk score curve (Figure 4C) demonstrates a clear distinction between IA patients and controls, with IA patients exhibiting significantly higher risk scores. This robust discrimination highlights the potential of the pyroptosis-related gene signature as a diagnostic tool.

### 3.5 Validation of PRGs as diagnostic biomarkers in IA

**Figure 5:**
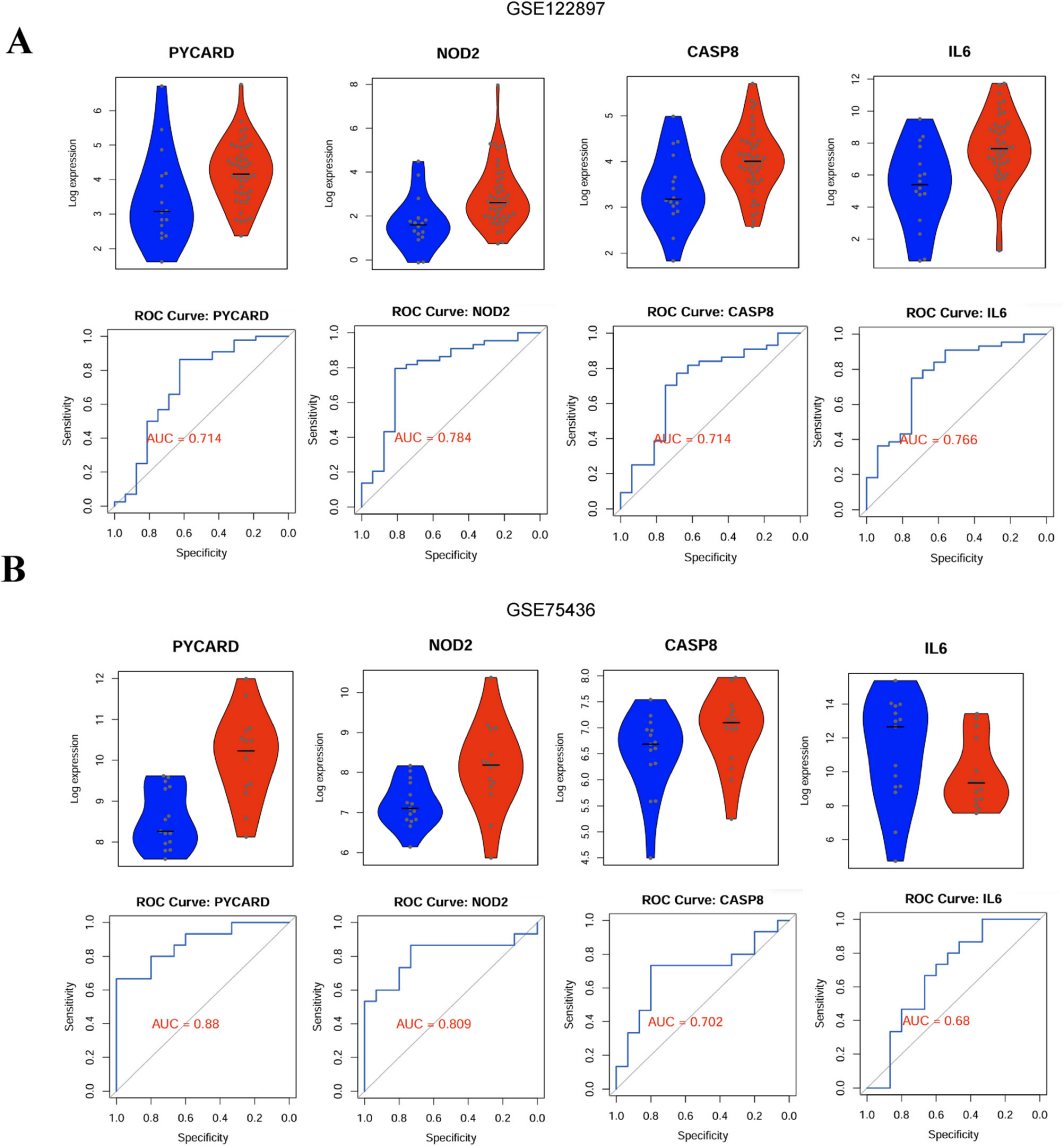
Validation of PRGs as diagnostic biomarkers in IA. (A) Violin plots showing the expression of PRGs (PYCARD, NOD2, CASP8, IL6) in IA versus control samples. ROC curves assessing diagnostic accuracy, with AUC values of 0.714 (PYCARD), 0.784 (NOD2), 0.714 (CASP8), and 0.766 (IL6). (B) Validation in an independent cohort, with improved AUC values for PYCARD (0.88) and NOD2 (0.809).

We evaluated the diagnostic potential of four heat protein deposition-related genes (PYCARD, NOD2, CASP8, and IL6) in IA. In the primary dataset (Figure 5A), all four genes exhibited significantly higher expression in IA samples compared to controls. ROC curve analysis demonstrated moderate diagnostic accuracy, highlighting their potential in distinguishing IA patients from controls. To ensure robustness, these findings were validated using an independent dataset (Figure 5B). Consistent with the primary analysis, all four genes were significantly upregulated in IA samples. ROC analysis in this dataset showed improved diagnostic performance, further reinforcing their relevance as diagnostic biomarkers. These results highlight PYCARD and NOD2 as particularly strong diagnostic candidates due to their consistent high expression and robust AUC values across datasets. Overall, this analysis supports the potential of these heat protein deposition-related genes as reliable biomarkers for IA diagnosis, laying a foundation for clinical translation.

### 3.6 Immune cell infiltration and PRGs in IA

**Figure 6:**
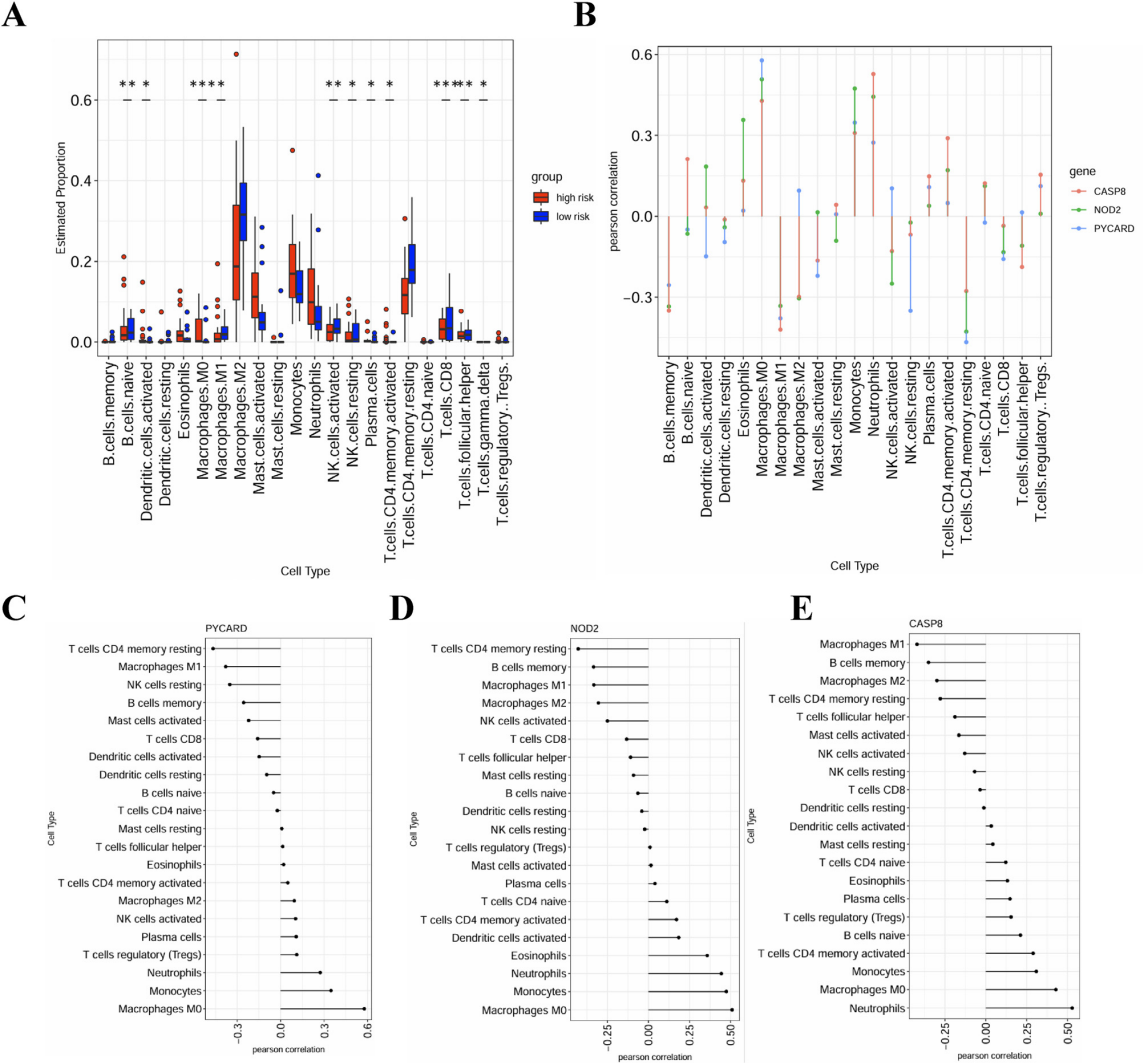
Immune cell infiltration and PRG correlation in IA. (A) Boxplot showing immune cell proportions in high-risk versus low-risk IA groups, highlighting differences in M1/M2 macrophages and activated dendritic cells. (B) Correlation plot showing significant associations between PRG expression (CASP8, NOD2, PYCARD) and immune cell types. (C-E) Forest plots showing Pearson correlation coefficients between PRGs and immune cells, emphasizing their role in immune modulation in IA.

To understand the role of PRGs in IA, we analyzed immune cell infiltration and its association with PRG expression. A comparison of immune cell proportions between high-risk and low-risk IA groups (Figure 6A) revealed significantly higher levels of activated dendritic cells, M1 macrophages, and M2 macrophages in the high-risk group. Correlation analysis highlighted clear associations between PRGs (PYCARD, NOD2, and CASP8) and immune cell infiltration (Figure 6B). PYCARD and NOD2 showed strong positive correlations with memory CD4+ T cells and M2 macrophages, indicating their roles in adaptive immunity and anti-inflammatory pathways. In contrast, CASP8 was closely associated with M1 macrophages and neutrophils, reflecting its involvement in pro-inflammatory responses. Building on the observed differences in immune cell infiltration between high-risk and low-risk IA groups, single-gene correlation analysis (Figure 6C–E) provided further insights into the specific immune regulatory roles of PYCARD, NOD2, and CASP8. PYCARD correlated with memory CD4+ T cells and activated NK cells, indicating its role in innate and adaptive immune activation. NOD2 was linked to memory B cells and M2 macrophages, highlighting its anti-inflammatory function in maintaining immune homeostasis. CASP8 showed strong associations with M1 macrophages and neutrophils, emphasizing its pro-inflammatory role in IA progression. These findings suggest that PRGs shape a complex immune landscape by balancing pro-inflammatory and anti-inflammatory responses, which is critical to IA pathophysiology.

### 3.7 Validation of genes upregulation and caspase 8 activation in IA samples

**Figure 7:**
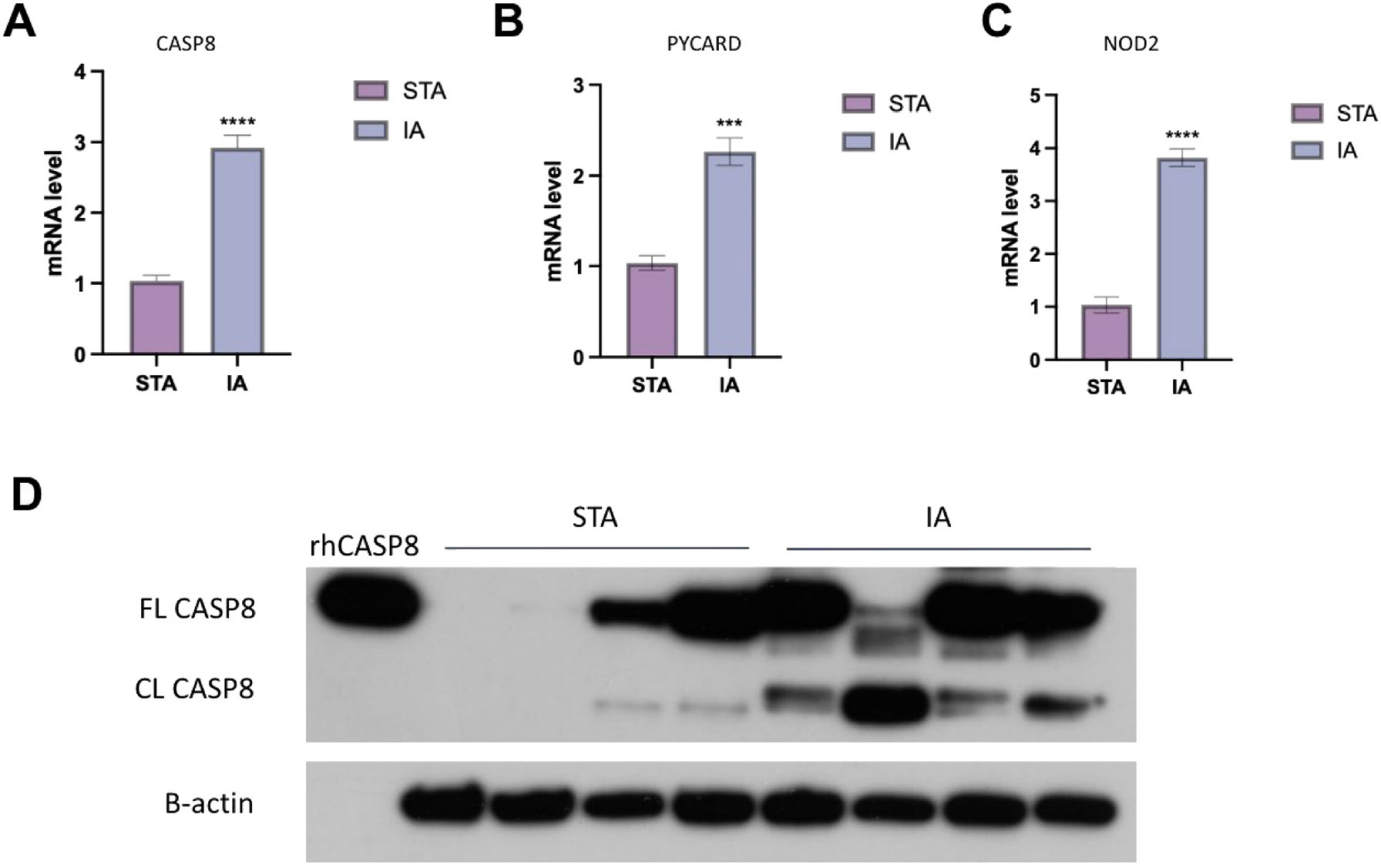
Validation of candidate diagnostic biomarkers in IA and STA samples. (A–C) Quantitative real-time PCR analysis of CASP8, PYCARD, and NOD2 mRNA levels in STA and IA tissue samples. All three genes were significantly upregulated in IA tissues compared with STA. (D) Western blot analysis of CASP8 protein expression. FL CASP8: full-length caspase-8; CL CASP8: cleaved caspase-8. Recombinant human CASP8 protein (rhCASP8) was used as a positive control. B-actin was used as a loading control. Data are shown as mean ± SD; ****p < 0.0001, ***p < 0.001.

To validate the expression levels of candidate diagnostic biomarkers, we performed quantitative real-time PCR and Western blot analysis using IA and STA tissue samples. The qPCR results showed that the mRNA expression levels of CASP8, PYCARD, and NOD2 were significantly upregulated in IA tissues compared with STA controls (Figure 7A–C). Among them, CASP8 and NOD2 showed a more than twofold increase, while PYCARD was moderately elevated.

Consistently, Western blot analysis demonstrated that the protein level of full-length CASP8 (FL CASP8) was notably increased in IA samples compared with STA tissues (Figure 7D). In addition, cleaved CASP8 (CL CASP8, P41/43) fragments were also clearly detected in IA samples, suggesting the activation of CASP8 in intracranial aneurysm tissues. Recombinant human CASP8 protein (rhCASP8) was used as a positive control. These results indicate that CASP8 is not only transcriptionally upregulated but also proteolytically activated in IA tissues. The consistent upregulation and activation of CASP8 in IA tissues may play a central role in disease progression by amplifying inflammatory responses and modulating endothelial cell function, both of which are critical in aneurysm pathophysiology. Such findings highlight CASP8 as a potential therapeutic target for managing IA. Additionally, we examined the expression and activation of GSDMD, but found that GSDMD was not activated in IA samples. Therefore, while the activation of CASP8 may be associated with pyroptosis, the role of GSDMD in IA appears to be insignificant, providing new insights into the pyroptosis mechanism in IA.

## 4 Discussion

IA is a devastating cerebrovascular disease characterized by the localized dilation of arterial walls, which often leads to rupture and subarachnoid hemorrhage. Despite significant advancements in diagnostic imaging and clinical management, the molecular mechanisms underlying IA remain insufficiently understood [29]. Our study aimed to investigate the involvement of pyroptosis, a form of programmed inflammatory cell death, in the pathogenesis of IA. We identified and validated a pyroptosis-related gene signature using bioinformatics and machine learning approaches, which provides novel insights into the molecular underpinnings of IA and potential biomarkers for early detection and targeted intervention.

The differential expression analysis revealed key PRGs, including CASP8, NOD2, and PYCARD, which were strongly associated with IA progression. These genes have been implicated in various inflammatory processes, and their dysregulation may contribute to the vascular remodeling observed in IA [30]. Pyroptosis, mediated by inflammasomes and PRGs, promotes the release of pro-inflammatory cytokines and alarmins [31], which can exacerbate vascular damage and further drive the pathological progression of IA [12], 32]. By identifying these genes, we have highlighted their potential role as diagnostic and prognostic biomarkers for IA.

Machine learning models were used to refine the predictive capacity of these PRGs, resulting in a robust gene signature capable of distinguishing high-risk IA patients from low-risk controls. The validation of this gene signature across independent cohorts confirmed its diagnostic and prognostic potential, demonstrating its ability to stratify IA patients based on risk. This provides a foundation for the development of more accurate and personalized diagnostic tools for IA, enabling earlier detection and better risk management for patients.

Additionally, immune cell infiltration analysis and correlation studies provided further insights into the interaction between PRGs and the immune microenvironment in IA. High-risk IA samples exhibited elevated levels of immune cells, such as macrophages and activated dendritic cells, suggesting that pyroptosis not only drives inflammation but also plays a role in immune cell recruitment and activation [33], 34]. The immune landscape in IA appears to be dynamic and complex, with distinct PRGs influencing both pro-inflammatory and anti-inflammatory pathways [35]. Our findings indicate that PRGs like CASP8 and PYCARD may contribute to pro-inflammatory responses, while NOD2 is more involved in anti-inflammatory mechanisms. This balance of immune responses could be crucial for understanding IA progression and identifying potential therapeutic targets.

The role of pyroptosis in IA highlights a potential new avenue for therapeutic intervention. By targeting key PRGs, it may be possible to modulate the inflammatory response and vascular remodeling associated with IA, potentially preventing aneurysm rupture and improving patient outcomes. Moreover, understanding the interplay between pyroptosis and immune dysregulation in IA could provide new strategies for immunomodulatory therapies that specifically target the underlying molecular mechanisms driving disease progression.

CASP8 is a key finding in our study, as its activation is involved in cell death and inflammatory responses [36]. CASP8 participates in pyroptosis and may play an important role in inflammation and vascular remodeling, promoting the progression of aneurysms. However, understanding the upstream activation pathways of CASP8 remains a challenge. Key signaling molecules, such as FADD (Fas-associated protein with death domain), are known to play a critical role in activating CASP8 through death receptors. FADD interacts with Fas receptors and other death receptors to form a complex, recruiting and activating CASP8 [37]. In the context of IA, inflammatory stimuli, such as inflammasome activation or other cellular stress responses, may trigger CASP8 activation through FADD or other related proteins. Although the precise mechanisms remain unclear, exploring the role of FADD and other upstream activators in CASP8 activation may provide valuable insights into the inflammatory processes driving aneurysm formation and rupture in IA, and this remains an important direction for future research.

Despite the promising findings, several limitations must be acknowledged. First, the use of publicly available datasets for analysis may introduce inherent biases, and additional validation in clinical settings is necessary. Furthermore, the precise functional roles of the identified PRGs in IA need to be further explored through other experimental studies, including gene knockdown and overexpression models. Future research should also focus on elucidating the precise mechanisms by which these genes influence immune responses and vascular remodeling in IA. Finally, the application of this gene signature in clinical practice would require prospective studies to assess its utility in guiding personalized treatment strategies and improving patient outcomes.

In conclusion, this study offers a comprehensive analysis of PRGs in IA, providing a novel molecular perspective on its pathogenesis. The identified gene signature serves as a potential biomarker for early detection and risk stratification. The discovery of caspase 8 activation opens new avenues for targeted therapeutic interventions. By exploring the relationship between pyroptosis and immune dysregulation, this study contributes to the growing understanding of IA and paves the way for the development of innovative diagnostic and treatment strategies aimed at improving patient care.

## 5 Conclusions

Our study identified and validated a pyroptosis-related gene signature in IA, shedding light on the molecular mechanisms behind IA pathogenesis. Bioinformatics analysis revealed key PRGs (CASP8, NOD2, and PYCARD) strongly linked to IA progression. Machine learning approaches further refined these genes into a predictive signature with diagnostic and prognostic potential, validated across independent cohorts. Our analysis also highlighted the complex interaction between these PRGs and the immune microenvironment, with elevated immune cell levels in high-risk IA samples. We also validated the upregulation of the PRGs and the activation of caspase 8 by qPCR and WB. These findings emphasize the crucial role of pyroptosis in inflammatory processes contributing to vascular remodeling in IA. Our study offers new opportunities for more effective clinical interventions.
